# Heavy Chain Disease with Cystic Lung Disease Presenting as Recurrent Spontaneous Pneumothorax in a Young Adult

**DOI:** 10.15388/Amed.2025.32.2.15

**Published:** 2025-12-30

**Authors:** Konstantinos Dodos, Tsampika Vasileia Kalamara, Vasiliki Epameinondas Georgakopoulou

**Affiliations:** 1 Laboratory of Physiology, School of Medicine, Aristotle University of Thessaloniki, Thessaloniki, Greece; 2 Laboratory of Physiology, School of Medicine, Aristotle University of Thessaloniki, Thessaloniki, Greece; 3 Department of Pathophysiology, Laiko General Hospital; National and Kapodistrian University of Athens, Athens, Greece

**Keywords:** heavy chain disease, γ-heavy chain disease, cystic lung disease, spontaneous pneumothorax, monoclonal gammopathy, plasma cell disorders, Sunkiųjų grandinių liga, γ-SGL sunkiųjų grandinių liga, cistinė plaučių liga, spontaninis pneumotoraksas, monokloninė gamopatija, plazminių ląstelių sutrikimai

## Abstract

Heavy chain diseases (HCDs) are rare B-cell/plasma cell disorders characterized by secretion of truncated immunoglobulin heavy chains without light chains. Pulmonary involvement has been described but is typically limited to interstitial or infiltrative patterns; whereas, cystic lung disease is exceptionally rare. We report a 23-year-old previously healthy male who presented with recurrent spontaneous pneumothoraces over a two-year period. High-resolution computed tomography revealed numerous bilateral thin-walled cysts with upper-lobe predominance, with several of these abutting the pleural surface. Laboratory evaluation demonstrated a discrete monoclonal spike on serum protein electrophoresis, and immunofixation confirmed an isolated IgG heavy chain without light chains, consistent with γ-heavy chain disease (γ-HCD). Bone marrow biopsy showed a mild increase in plasma cells (5–10%) without overt malignancy, and alternative causes of cystic lung disease, including Birt-Hogg-Dubé syndrome, autoimmune disease, α1-antitrypsin deficiency, and HIV, were excluded. This case highlights γ-HCD as a rare cause of diffuse cystic lung disease with recurrent pneumothorax, expanding the pulmonary spectrum of heavy-chain dyscrasias. The radiographic overlap with light-chain deposition disease emphasizes the need to include monoclonal gammopathies in the differential diagnosis of unexplained cystic lung disease. Recognition of γ-HCD in this context is clinically important, as it may precede lymphoproliferative malignancy and mandates careful longitudinal surveillance.

## Abstract (it)

Sunkiųjų grandinių ligos (SGL) – retos B limfocitų / plazminių ląstelių kilmės sutrikimų formos, kurioms būdinga sutrumpėjusių imunoglobulinų sunkiųjų grandinių sekrecija be lengvųjų grandinių. Plaučių pažeidimas dažniausiai pasireiškia intersticiniais ar infiltraciniais pokyčiais; cistinė plaučių liga išskirtinai reta. Sveikam 23 metų vyrui dvejus metus kartojosi spontaniniai pneumotoraksai. Aukštos raiškos kompiuterinė plaučių tomografija parodė esant daugybines abipuses plonasienių cistų struktūras – dominavo viršutinės skiltys, kai kurios iš jų siekė pleurą. Laboratoriniai tyrimai atskleidė diskretinį monokloninį piką serumo baltymų elektroforezėje, o imunofiksacija patvirtino izoliuotą IgG sunkiąją grandinę be lengvųjų grandinių, kas atitiko γ-sunkiųjų grandinių ligos (γ-SGL) diagnozę. Atlikus kaulų čiulpų biopsiją nustatyta nežymi plazminių ląstelių proliferacija (5–10 %), tačiau nebuvo piktybiškumo požymių. Kitos galimos cistinės plaučių ligos priežastys (Birt-Hogg-Dubé sindromas, autoimuninės ligos, α1- antitripsino stoka, ŽIV infekcija) buvo atmestos. Šiuo atveju pabrėžiama, kad γ-SGL yra reta difuzinės cistinės plaučių ligos ir pasikartojančio spontaninio pneumotorakso priežastis. Ligą nustatyti šioje klinikinėje situacijoje yra itin svarbu, nes ji gali būti priešpiktybinės limfoproliferacinės būklės išraiška ir reikia nuolatinės stebėsenos.

## Introduction

*Heavy Chain Diseases* (HCDs) are rare B-cell/plasma cell lymphoproliferative disorders characterized by secretion of truncated monoclonal immunoglobulin heavy chains that lack associated light chains due to structural deletions – often involving the CH1 domain – which permit abnormal heavy chains to escape endoplasmic reticulum retention and be secreted without pairing to light chains [1–3]. Three clinicopathologic variants are recognized based on heavy chain class, specifically, α (IgA), γ (IgG), and μ (IgM), with γ-HCD considered the most frequently reported in contemporary series, though overall fewer than a few hundred cases have been documented globally since the first description in 1964 [1–4]. Clinical presentation is heterogeneous and may resemble lymphoma more than myeloma, with constitutional symptoms, lymphadenopathy, hepatosplenomegaly, autoimmune phenomena, and, occasionally, overlapping lymphoid neoplasms; a standardized treatment approach is lacking, and therapy is typically individualized [1,3–6].

Pulmonary involvement in HCD is uncommon, but has been described across case reports and small series, variably manifesting as interstitial infiltrates, lymphoid interstitial pneumonia-like changes, or nodular disease; truly cystic parenchymal disease appears exceptional in γ-HCD [4,7]. By contrast, cystic lung disease is more classically linked to *Light Chain Deposition Disease* (LCDD) and related monoclonal immunoglobulin deposition disorders, where multiple thin-walled cysts with or without nodules can be seen on high-resolution CT and may coexist with autoimmune conditions such as Sjögren’s syndrome [8–10]. Importantly, diffuse cystic lung diseases (DCLDs) are an established risk factor for secondary spontaneous pneumothorax, and recurrence rates are high across entities such as lymphangioleiomyomatosis, Birt-Hogg-Dubé syndrome, pulmonary Langerhans cell histiocytosis, and lymphoid interstitial pneumonia [11–13]. Therefore, in young patients with recurrent spontaneous pneumothorax and diffuse cystic changes, a structured diagnostic algorithm that integrates HRCT patterns, clinical context (sex, smoking status, syndromic features), and targeted laboratory testing is critical to narrow the differential and identify less typical causes – including monoclonal gammopathies – when initial evaluation is unrevealing [11,12,14].

Against this background, reports of γ-HCD associated with pulmonary disease underscore the need to consider paraproteinemic disorders in the differential diagnosis of unexplained cystic lung disease, particularly when serum studies detect an isolated heavy chain without kappa or lambda light chains [4,7]. Recognition is clinically relevant because HCD may evolve alongside or precede overt lymphoproliferative malignancy, and management decisions (observation versus immunochemotherapy or anti-CD20–based regimens) depend on the tempo, symptom burden, and associated conditions [1,3,5,6]. The present case adds to the limited literature by highlighting cystic lung disease with recurrent pneumothorax as a rare pulmonary presentation of γ-HCD in a young adult, thus reinforcing the value of a multidisciplinary approach that bridges pulmonology, hematology, radiology, and pathology.

## Case Presentation

A 23-year-old Caucasian male with no prior medical history, no tobacco or illicit drug use, and no known occupational exposures presented with acute-onset right-sided pleuritic chest pain and dyspnea. On admission, vital signs revealed tachycardia (110 bpm), tachypnea (24 breaths/min), and oxygen saturation of 92% on room air. Physical examination demonstrated markedly diminished breath sounds over the right hemithorax with hyperresonance to percussion.

**Figure 1:**
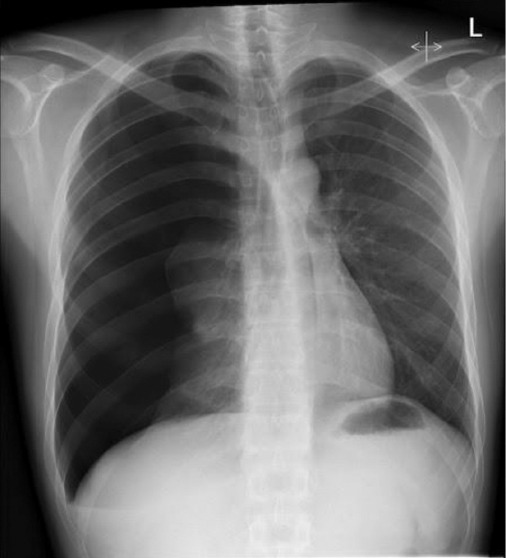
Chest radiograph of spontaneous pneumothorax

A portable chest radiograph confirmed a right-sided pneumothorax involving approximately 30% lung volume (Figure 1).

The patient underwent emergent tube thoracostomy with complete re-expansion and was discharged after an uneventful three-day hospitalization. During the subsequent 24 months, he experienced three further episodes of spontaneous pneumothorax – of those, two right-sided and one left-sided – each managed with tube thoracostomy.

**Figure 2:**
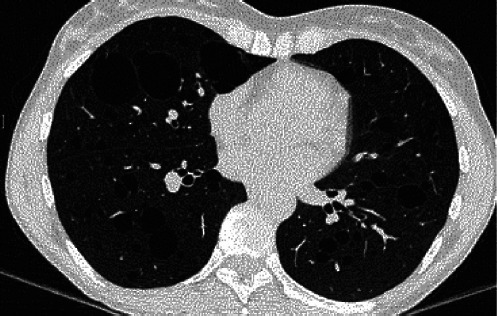
High-resolution computed tomography (HRCT) of the chest

Given the recurrence of spontaneous pneumothoraces in a young individual without the traditional risk factors, high-resolution computed tomography (HRCT) of the chest was performed. HRCT demonstrated numerous bilateral, thin-walled cystic lesions ranging from 5 mm to 2 cm, with an upper-lobe predominance and several cysts abutting the pleural surface, consistent with the likely sites of rupture. No interstitial infiltrates, parenchymal nodules, or mediastinal lymphadenopathy were identified (Figure 2).

The radiographic findings prompted further investigation for underlying cystic lung disease. Initial laboratory tests showed mild normocytic anemia with hemoglobin 11.2 g/dL (reference range 13.5–17.5 g/dL) but were otherwise within normal limits. Serum protein electrophoresis detected a discrete monoclonal spike in the gamma region (0.6 g/dL). Immunofixation electrophoresis confirmed the presence of an IgG heavy chain component in the absence of kappa or lambda light chains, establishing the diagnosis of γ-heavy chain disease (γ-HCD). A serum free light chain assay demonstrated kappa 12 mg/L (reference 3.3–19.4 mg/L), lambda 18 mg/L (reference 5.7–26.3 mg/L), with a normal kappa-to-lambda ratio.

Bone marrow biopsy revealed mildly increased plasma cells (5–10% of cellularity) without morphologic or immunophenotypic evidence of overt malignancy. Flow cytometry did not demonstrate a clonal B-cell population. Additional evaluations excluded alternative etiologies of diffuse cystic lung disease, including normal α1-antitrypsin levels (reference 90–200 mg/dL), negative HIV serology, and negative autoimmune markers (ANA, RF, ANCA). Germline testing for *FLCN* mutations associated with Birt-Hogg-Dubé syndrome was also negative. Given the exclusion of the more common etiologies of diffuse cystic lung disease, serum protein electrophoresis and immunofixation were performed in accordance with our institutional diagnostic algorithm for unexplained cystic lung disease in young patients. This testing revealed a monoclonal IgG heavy chain, which guided the subsequent hematologic evaluation and the final diagnosis of γ-heavy chain disease.

The patient remained under close hematologic and pulmonary follow-up for 18 months after diagnosis. During this period, there was no hematologic progression or development of lymphoproliferative disease, and the monoclonal spike remained stable. A follow-up HRCT at 12 months demonstrated no increase in the number or size of pulmonary cysts. After pleurodesis, no further pneumothorax episodes were recorded.

## Discussion

This case underscores an atypical pulmonary presentation of γ–heavy chain disease (γ-HCD): diffuse cystic lung disease complicated by recurrent, bilateral spontaneous pneumothoraces in a young man without the traditional risk factors. Pulmonary involvement in γ-HCD is reported but uncommon, and, when present, it more often resembles infiltrative or interstitial patterns rather than a cyst-predominant phenotype. Contemporary reviews and series emphasize the clinical heterogeneity of γ-HCD and its frequent association with autoimmune disease or lymphoproliferative neoplasms; however, cystic parenchymal disease and pneumothorax at presentation are not the defining features [15,16].

In the published literature on γ-HCD, lung manifestations include parenchymal infiltrates or secondary complications (for example, pulmonary hypertension responding to lenalidomide, or iatrogenic immunodeficiency-associated lymphoproliferative disease after methotrexate), but we were unable to identify prior reports in which diffuse cystic lung disease with recurrent pneumothorax was the sentinel presentation of γ-HCD [7,17]. This contrast is notable because cystic patterns are well documented in pulmonary LCDD, where thin-walled cysts (often with nodules) are typical and may progress to respiratory failure or necessitate transplantation. Our patient’s imaging therefore aligns radiographically with LCDD yet immunochemically fulfills criteria for γ-HCD, thus expanding the recognized pulmonary spectrum of heavy-chain dyscrasias [8,9,18–20].

Potential mechanisms for cyst formation in paraproteinemic lung disease include peribronchiolar or interstitial immunoglobulin deposition causing a ball-valve effect, small-airway remodeling with alveolar wall fragility, and traction from bronchiolar obstruction; these have been proposed and histologically supported in LCDD and may be extrapolated – albeit cautiously – to heavy-chain-predominant disorders [18,19]. Direct lymphoplasmacytic infiltration with architectural distortion is an alternative mechanism relevant to γ-HCD, particularly in cases with coexisting lymphoid neoplasia. The upper-lobe predominance in our patient could mimic pulmonary Langerhans cell histiocytosis radiographically, but the absence of typical nodules and the serologic profile argue against this, reinforcing the need for a paraproteinemia work-up in atypical cystic patterns [19,21].

Recurrent pneumothorax is a recognized complication across DCLDs; recurrence rates are high, and some patients experience multiple events. Our patient’s four pneumothoraces over two years are consistent with the recurrence burden described for DCLDs, which highlights the importance of early pleural-based risk stratification and consideration of definitive pleural interventions (e.g., pleurodesis) when cysts abut the pleura [12].

From a diagnostic standpoint, this case illustrates the value of integrating HRCT pattern recognition with targeted hematologic testing. A stepwise algorithm for cystic lung disease recommends pairing imaging phenotypes with the clinical context and serologic/genetic panels (including *FLCN* for Birt-Hogg-Dubé, HIV, autoimmune serologies, α1-antitrypsin, and – crucially here – serum protein electrophoresis with immunofixation). The detection of an IgG heavy-chain band without the associated light chains is pathognomonic for γ-HCD and should prompt bone-marrow assessment and longitudinal hematologic follow-up [13].

A key limitation of this report is the absence of histologic confirmation of lung involvement, as no lung biopsy or autopsy was performed. While the radiologic pattern and exclusion of alternative diagnoses support an association between γ-heavy chain disease and cystic lung disease, a definitive causal link cannot be established without tissue evidence of immunoglobulin deposition or lymphoplasmacytic infiltration. This limitation underscores the need for caution when interpreting the pulmonary findings and highlights the importance of future reports that include pathologic confirmation.

Management of γ-HCD is individualized, and it ranges from observation in indolent, pauci-symptomatic cases to immunochemotherapy when there is systemic involvement or symptomatic progression. Small series and case reports describe responses to rituximab-based regimens and immunomodulatory agents; more recently, anti-CD38-containing combinations have been reported, reflecting therapeutic borrowing from plasma-cell dyscrasias. In our patient, the low-level M-protein, absence of constitutional symptoms, and lack of overt lymphoma or organomegaly supported active surveillance, with pulmonary management focused on pneumothorax prevention and monitoring for progression [16,17].

Importantly, longitudinal follow-up over 18 months demonstrated stable hematologic findings and no radiologic progression of cystic lung disease. The absence of further pneumothorax episodes after pleurodesis supports the role of early pleural intervention in preventing recurrence in patients with cyst-abutting pleura.

## Conclusion

This case illustrates a highly unusual pulmonary manifestation of γ-heavy chain disease, presenting with diffuse cystic lung disease and recurrent spontaneous pneumothorax in a young adult. Although pulmonary involvement in HCD is rare and usually non-cystic, this report expands the recognized spectrum of disease and underscores the importance of considering paraproteinemic disorders in the differential diagnosis of unexplained cystic lung disease. Early recognition facilitates appropriate monitoring, informs risk of pneumothorax recurrence, and ensures timely hematologic follow-up given the potential for progression toward overt lymphoproliferative malignancy.
